# Supplementary material for: Characteristics, Patterns of Care and Predictive Geriatric Factors in Elderly Patients Treated for High-Grade IDH-Mutant Gliomas: A French POLA Network Study
Source: Cancers (Basel). 2022 Nov 9;14(22):5509. doi: 10.3390/cancers14225509 (PMC9688655; doi:10.3390/cancers14225509)
Supplement: Supplementary file 1 [file cancers-14-05509-s001.zip › cancers-1949010-suppl-figure.pdf]

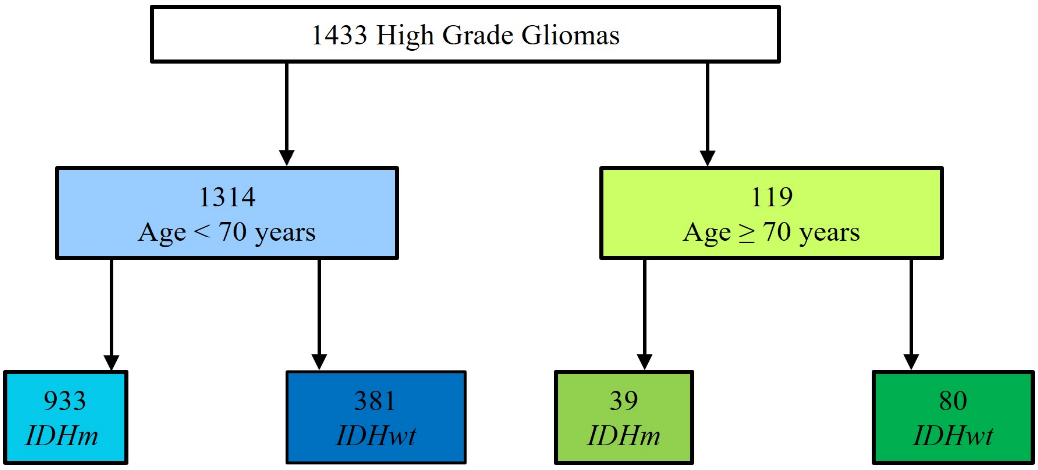

Figure S1. Patient repartition according to the age (< or ≥ 70 years) and the IDH mutation status in POLA cohort.

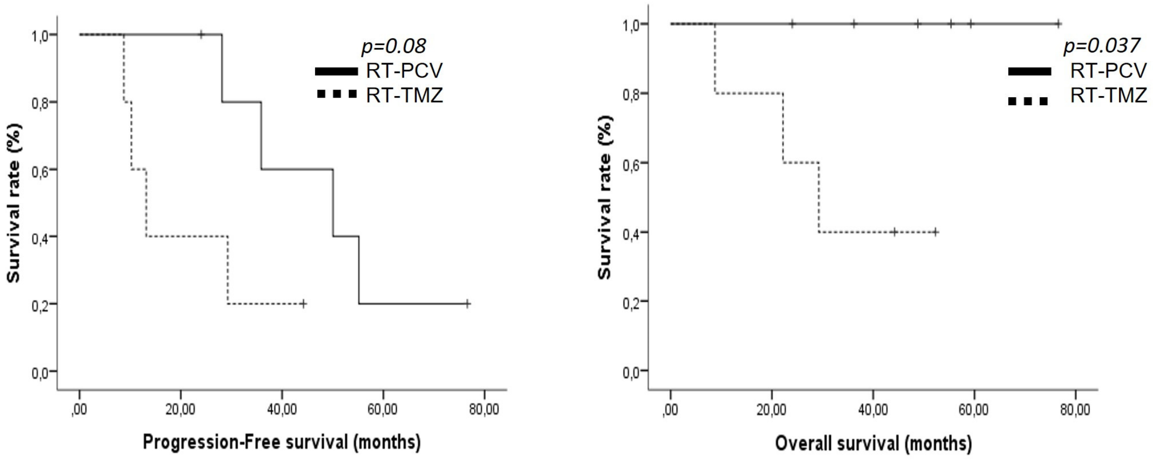

Figure S2. Progression free-survival (left) and overall survival (right) according to treatment groups in elderly patients with anaplastic oligodendroglioma. RT: radiotherapy; PCV: procarbazine, CCNU, Vincristine; TMZ: temozolomide.
