# Supplementary material for: Characteristics, Patterns of Care and Predictive Geriatric Factors in Elderly Patients Treated for High-Grade IDH-Mutant Gliomas: A French POLA Network Study
Source: Cancers (Basel). 2022 Nov 9;14(22):5509. doi: 10.3390/cancers14225509 (PMC9688655; doi:10.3390/cancers14225509)
Supplement: Supplementary file 1 [file cancers-14-05509-s001.zip › cancers-1949010-suppl-table.pdf]

**Supplementary Table S1. Baseline characteristics and treatment patterns in elderly patients *IDHm* HGG (Geriatric cohort, N=35) according to histological sub-type.**

| **Characteristics** | **Oligodendroglioma (n=25)** | **Astrocytoma (n=9)** |
|---|---|---|
| | ***N (% or range)*** | ***N (% or range)*** |
| **Age** (years, median, range) | 74.0 *(70.2 - 87.1)* | 73.1 *(70.2-83.1)* |
| **Gender** | | |
| Male | 14 *(56)* | 5 *(55.6)* |
| Female | 11 *(44)* | 4 *(44.4)* |
| **Symptoms at diagnosis** | | |
| Epilepsy | 11 *(44)* | 4 *(44.4)* |
| Cognitive disorders | 4 *(16)* | 3 *(33.3)* |
| **Neuro-imaging characteristics** | | |
| Contrast enhancement | 14 *(70)* | 8 *(100)* |
| Necrosis | 3 *(25)* | 3 *(33.3)* |
| **Histological characteristics** | | |
| Necrosis | 6 *(24)* | 9 *(100)* |
| Microvascular Proliferation | 20 *(80)* | 4 *(44.4)* |
| Ki 67 expression (mean, range) | 20 *(5-40)* | 10 *(1-25)* |
| **Post-operative KPS (median, range)** | 80 *(50-100)* | 70 *(50-100)* |
| **Steroids intake** | 11 *(55)* | 2 *(25)* |

| | | |
|---|---|---|
| **Extent of resection** | | |
| Gross total /Subtotal | 7 *(29.2)* | 2 *(22.2)* |
| Partial | 4 *(16.7)* | 1 *(11.1)* |
| Biopsy | 13 *(54.1)* | 6 *(66.7)* |
| **Adjuvant Treatment** | | |
| Wait and scan policy | 2 *(8)* | 0 |
| Radiotherapy alone | 1 *(4)* | 0 |
| Chemotherapy alone<br>TMZ<br>PCV | <br>6 *(24)*<br>4 *(16)* | <br>6 *(67)*<br>0 |
| RT-TMZ | 5 *(20)* | 3 *(33)* |
| RT-PCV | 6 *(24)* | 0 |
| Palliative care | 1 *(4)* | 0 |
| **Cognitive disorders** | | |
| Neuropsychological disorders[a] | 4 *(23.5)* | 3 *(37.5)* |
| **Mobility** | | |
| Get out without assistance | 13 *(81.3)* | 3 *(37.5)* |
| **Comorbidities** | | |
| Charlson's index ≥ 5 | 15 *(65.2)* | 8 *(88.9)* |
| Medications > 3 | 6 *(35.3)* | 3 *(33.3)* |
| **Nutrition** | | |

| | | |
|---|---|---|
| Anorexia | 2 *(12.5)* | 4 *(50)* |
| Weight loss | 7 *(41.2)* | 5 *(62.5)* |
| BMI < 21 | 4 *(22.2)* | 3 *(33.3)* |
| **Autonomy** | | |
| ADL < 6 | 3 *(27.3)* | 3 *(42.9)* |
| IADL < 4 | 6 *(54.5)* | 2 *(33.3)* |
| **Not a good self-reported state of health** | 2 *(16.7)* | 4 *(80)* |
| **G8 score estimation ≤ 14/17** | 10 *(58.8)* | 6 *(75)* |

KPS: Karnofsky Performance Scale; BMI: Body Mass Index; ADL: Activities of Daily Living; IADL: Instrumental Activities of Daily Living.
[a] Neuropsychological disorder assessed by G8 score.

**Supplementary Table S2. Treatment feasibility and toxicity in elderly patients according to chemotherapy adjuvant protocol (N=29).** TMZ: temozolomide; PCV: procarbazine, CCNU, vincristine.

| | **TMZ N (%)** | **PCV N (%)** |
|---|---|---|
| ***Number of patients*** | 19 | 10 |
| **Number of cycles:** ≥ 6 (TMZ); =6 (PCV) | 13 *(72.3)* | 5 *(50)* |
| **Dose reduction at treatment initiation** | 7 *(41.1)* | 5 *(62.5)* |
| **Dose reduction maintaining (without AE)** | 3 *(18.8)* | 1 *(12.5)* |
| **Dose reduction during treatment due to AE** | 3 *(18.8)* | 7 *(87.5)* |
| **Treatment interruption due to AE** | 2 *(12.5)* | 6 *(75)* |
| **AE grade ≥ 3** | 3 *(16)* | 2 *(20)* |

TMZ: Temozolomide; PCV: Procarbazine, CCNU, Vincristine; AE: Adverse Events grading with Criteria scale for adverse events version 4.0.

**Supplementary Table S3. Treatment toxicity in elderly patients according to chemotherapy adjuvant protocol (N=29).**

| | TMZ (N=19) N (%) | | PCV (N=10) N (%) | |
|---|---|---|---|---|
| *CTCAE* | **Grade 1 or 2** | **Grade ≥3** | **Grade 1 or 2** | **Grade ≥3** |
| **Haemoglobin decrease** | 1 *(5.2)* | - | - | - |
| **Platelet count decrease** | 3 *(15.8)* | 1 *(5.2)* | 2 *(20)* | 1 *(10)* |
| **Neutropenia** | 1*(5.2)* | - | 2 *(20)* | - |
| **Febrile neutropenia** | - | - | - | - |
| **Urinary infection** | - | - | 1 *(10)* | - |
| **Lymphopenia** | - | 1 *(5.2)* | - | - |
| **Asthenia/Fatigue** | 8 *(42.1)* | - | 3 *(30)* | 3 *(30)* |
| **Anorexia/Weight loss** | 3 *(15.8)* | - | - | 1 *(10)* |
| **Constipation** | 1 *(5.2)* | - | 1 *(10)* | - |
| **Nausea/Vomiting** | 2 *(10.5)* | - | 1 *(10)* | - |
| **Cutaneous AE (dermatitis, allergic rash, alopecia)** | 1 *(5.2)* | - | 1 *(10)* | - |
| **Neuropathy** | - | - | 2 *(20)* | - |

| | | | | |
|---|---|---|---|---|
| **Thromboembolic event** | - | - | - | - |
| **Liver enzyme elevation** | - | - | - | - |

TMZ: Temozolomide; PCV: Procarbazine, CCNU, Vincristine; AE: Adverse Events grading with Criteria scale for adverse events version 4.0.

**Supplementary Table S4. Predictive factors of treatment toxicity in elderly *IDH* mutated high-grade glioma cohort (N=34).**

| | **AE grade ≥ 3** | **Treatment interruption due to toxicity** |
|---|---|---|
| | ***p-value*** | ***p-value*** |
| **G8 score (items)** | | |
| Anorexia | 0.690 | 0.279 |
| Weight loss | 0.082 | 0.174 |
| Mobility | 0.362 | 0.047 |
| Neuropsychological disorders | 0.380 | 0.490 |
| BMI | 0.290 | 0.115 |
| Number of medications > 3 | 1.000 | 0.631 |
| Self-reported state of health | 0.004 | 0.443 |
| Age ranges | 1.000 | 0.234 |
| **Autonomy** | | |
| ADL | 0.268 | 0.245 |
| IADL | 0.538 | 0.231 |
| **Charlson's index score** | 1.000 | 1.000 |
| **Post-operative KPS** | 0.013 | 0.529 |

AE: Adverse events grading with Criteria scale for adverse events version 4.0; BMI: Body Mass Index; ADL: Activities of Daily Living, IADL: Instrumental Activities of Daily Living, KPS: Karnofsky Performance Scale.
